# Synaptic Plasticity and Memory Retention in ZnO–CNT Nanocomposite Optoelectronic Synaptic Devices

**DOI:** 10.3390/ma18102293

**Published:** 2025-05-15

**Authors:** Seung Hun Lee, Dabin Jeon, Sung-Nam Lee

**Affiliations:** 1 Department of IT & Semiconductor Convergence Engineering, Tech University of Korea, Siheung 15073, Republic of Korea; 2 Department of Semiconductor Engineering, Tech University of Korea, Siheung 15073, Republic of Korea

**Keywords:** ZnO, sol–gel, carbon nanotube, optoelectronic, synapse, neuromorphic

## Abstract

This study presents the fabrication and characterization of ZnO–CNT composite-based optoelectronic synaptic devices via a sol–gel process. By incorporating various concentrations of CNTs (0–2.0 wt%) into ZnO thin films, we investigated their effects on synaptic behaviors under ultraviolet (UV) stimulation. The CNT addition enhanced the electrical and optical performance by forming a p–n heterojunction with ZnO, which promoted charge separation and suppressed recombination. As a result, the 1.5 wt% CNT device exhibited the highest excitatory postsynaptic current (EPSC), improved paired-pulse facilitation, and prolonged memory retention. Learning–forgetting cycles revealed that repeated stimulation reduced the number of pulses required for relearning while extending the forgetting time, mimicking biological memory reinforcement. Energy consumption per pulse was estimated at 16.34 nJ, suggesting potential for low-power neuromorphic applications. A 3 × 3 device array was also employed for visual memory simulation, showing spatially controllable and stable memory states depending on CNT content. To support these findings, structural and optical analyses were conducted using scanning electron microscopy (SEM), UV-visible absorption spectroscopy, photoluminescence (PL) spectroscopy, and Raman spectroscopy. These findings demonstrate that the synaptic characteristics of ZnO-based devices can be finely tuned through CNT incorporation, providing a promising pathway for the development of energy-efficient and adaptive optoelectronic neuromorphic systems.

## 1. Introduction

As Moore’s Law and Dennard scaling approach their physical and technological limitations, traditional von Neumann architectures are increasingly challenged in achieving high computational performance with low energy consumption [1]. In response to these limitations, neuromorphic computing, inspired by the structure and function of biological neural networks, has emerged as a promising paradigm for next-generation artificial intelligence (AI) hardware [2]. By integrating sensing, memory, and computation within a unified architecture, neuromorphic systems offer real-time data processing with significantly reduced power consumption [3]. To enable such systems, artificial synaptic devices that mimic biological synapse behaviors—such as learning, forgetting, and memory retention—are critical components [4]. While various electronic synaptic devices based on memristors, ferroelectrics, and 2D materials have been widely studied, optoelectronic synaptic devices are gaining considerable attention [5]. Their ability to utilize light as a presynaptic input allows for additional functionalities including wavelength-dependent selectivity, non-contact control, and seamless integration with vision-based neuromorphic systems [6,7]. These devices emulate synaptic plasticity by modulating the excitatory postsynaptic current (EPSC) in response to optical stimuli, thereby reproducing potentiation and depression dynamics found in biological systems [8]. To construct such light-responsive synapses, a wide range of materials has been explored—including oxide semiconductors (e.g., ZnO, In_2_O_3_), two-dimensional materials (e.g., MoS_2_, WS_2_), and organic–inorganic hybrids [9].

Among these, zinc oxide (ZnO) has emerged as a particularly attractive candidate due to its wide bandgap (3.37 eV), high sensitivity to UV light, chemical stability, and cost-effectiveness [10]. Additionally, ZnO is compatible with diverse thin-film deposition methods such as sputtering, chemical vapor deposition, atomic layer deposition, and sol–gel processing [11,12,13,14]. While CVD and ALD generally provide higher material purity and lower defect densities due to their vapor-phase precision, they require high temperatures, vacuum systems, and complex process control. Sputtering offers good film uniformity but often involves energetic plasma damage. In contrast, the sol–gel method was selected owing to its distinct advantages, including low-temperature processing, low-cost fabrication, large-area scalability, and microstructural tunability through precursor chemistry and thermal treatment [15]. Moreover, recent computational and modeling studies of nanocomposites, particularly those involving CNTs or nanowires, have demonstrated how interfacial stress and defect modulation can tune optical and electronic properties [16,17], supporting the importance of material design strategies in ZnO-based neuromorphic systems. Importantly, sol–gel-derived ZnO films inherently contain grain boundaries and native point defects, such as oxygen vacancies and zinc interstitials, which play a critical role in enhancing persistent photoconductivity (PPC)—a key mechanism enabling long-term synaptic memory in optoelectronic devices. However, these same defects also limit electrical conductivity, necessitating further material engineering [18,19,20]. To address this, carbon nanotubes (CNTs) were incorporated into the ZnO matrix to form nanocomposite thin films. CNTs possess high electrical and thermal conductivity, excellent mechanical strength, and a large surface-to-volume ratio [21]. Their integration not only improves electrical pathways but also induces nanowrinkle structures on the film surface during the drying and annealing process due to compressive stress. For example, CNT–ZnO nanocomposite photodetectors have demonstrated enhanced UV responsivity through defect engineering and surface modulation [22]. In addition, recent studies have shown that the formation of nanowrinkle structures in sol–gel-derived ZnO films can be strongly influenced by the soft-bake temperature, with direct implications for improving device performance. Specifically, controlled wrinkle formation has been exploited to enhance light extraction in LEDs [23] and to improve light absorption and sensitivity in photodetectors [24]. These wrinkle features enlarge the effective surface area and enhance light–matter interaction, thus facilitating oxygen adsorption/desorption dynamics under UV illumination [25]. Under such stimulation, ZnO–CNT composite films exhibit improved EPSC generation owing to more efficient carrier generation and enhanced interfacial charge transport. Furthermore, p-CNT/n-ZnO heterojunctions promote charge separation and suppress recombination [26]. However, beyond optimal CNT concentration, excessive CNT loading leads to aggregation and the formation of additional interfacial defects, which can degrade device performance. Interestingly, higher CNT content also contributes to the formation of deep-level trap states, which enhances the PPC effect, thereby extending synaptic retention and enabling long-term memory characteristics [27]. Systematic evaluation in this study revealed that a CNT concentration of 1.5 wt% yields the most favorable synaptic performance, including the highest EPSC, the longest forgetting time, and the most stable learning–forgetting cycles. These findings demonstrate the effectiveness of ZnO–CNT nanocomposite films as UV-responsive active layers in optoelectronic synaptic devices and highlight their potential for low-power, light-driven neuromorphic systems capable of in-sensor computing and intelligent memory applications.

## 2. Materials and Methods

Optoelectronic synaptic devices with an Al/ZnO–CNT/Al structure were fabricated by applying a sol–gel solution onto a sapphire substrate, followed by thin film formation through spin coating, and subsequent thermal evaporation of the electrode. Initially, the sapphire substrate was cleaned to ensure a high-quality and uniform sol–gel thin film. Organic contaminants were removed by ultrasonically cleaning the substrate sequentially with ethanol, isopropyl alcohol, and deionized water for 10 min each. After substrate cleaning, the prepared sol–gel solution was uniformly applied to the substrate. The sol–gel solution was prepared using zinc acetate dihydrate (ZAD), ethylene glycol monomethyl ether (2ME) as the solvent, and monoethanolamine (MEA) as the stabilizer. First, 0.025 mol of ZAD was added to a beaker, followed by 0.20 mol of 2ME, and the mixture was stirred until the ZAD completely dissolved. Second, 0.025 mol of MEA was added to the solution, which was stirred for one hour until it became transparent. The solution was then sealed in an airtight container and aged at room temperature for 24 h. CNT solutions were added to the sol–gel solution at varying concentrations of 0, 0.5, 1, 1.5, and 2.0 wt% to form samples with different CNT contents. The resulting sol–gel solution was deposited on the substrate using spin coating at 6000 revolutions per minute (RPM) for 30 s. The samples underwent soft baking on a hot plate at 150 °C for 5 min to improve film adhesion and remove residual solvents. A subsequent thermal treatment at 800 °C for 60 min in a furnace was performed to enhance the crystallinity and reduce dislocation of the ZnO thin films. Finally, a 50 nm-thick Al electrode was thermally evaporated on the ZnO–CNT thin film to complete the Al/ZnO–CNT/Al optoelectronic synaptic device fabrication.

Various methods and analytical tools were employed to characterize the surface, crystallographic, optical, and electrical properties of the ZnO–CNT nanocomposite films. Scanning Electron Microscopy (SEM, Coxem, Daejeon, Republic of Korea) was utilized to analyze the surface morphology and microstructure. The optical properties, including the bandgap, were determined using UV-visible absorption (Thermo Fisher Scientific, Evolution 300, Waltham, MA, USA) and photoluminescence (PL) measurements (Dongwoo, Seoul, Republic of Korea). Crystallographic properties were evaluated using Raman spectroscopy (Dongwoo, Seoul, Republic of Korea). Electrical performance was assessed by measuring both dark current and UV-induced photocurrent under 365 nm of UV illumination provided by a UV LED source using an HP4155A parameter analyzer (Santa Rosa, CA, USA). The optoelectronic synaptic behavior was characterized by recording the EPSC at a fixed bias of 1.0 V using the same analyzer. Optical potentiation was observed upon UV stimulation, resulting in an increase in the EPSC, followed by a natural depression as the EPSC gradually decreased after the UV source was turned off. The learning and forgetting processes were repeated twice, with the maximum EPSC defined as 100%, and the forgetting ratios evaluated at intervals of 700 and 1400 s. The visual memory images were generated by smoothly adjusting the color in proportion to the reduced size. This approach simulated gradual memory fading and was similarly applied to the second learning–forgetting process. The resulting images were implemented in a 3 × 3 array of cells.

## 3. Results and Discussion

### 3.1. Structural, Surface Morphological, and Optical Characterization of ZnO–CNT (0–2.0 wt%) Thin Films on Sapphire Substrates

**Figure 1:**
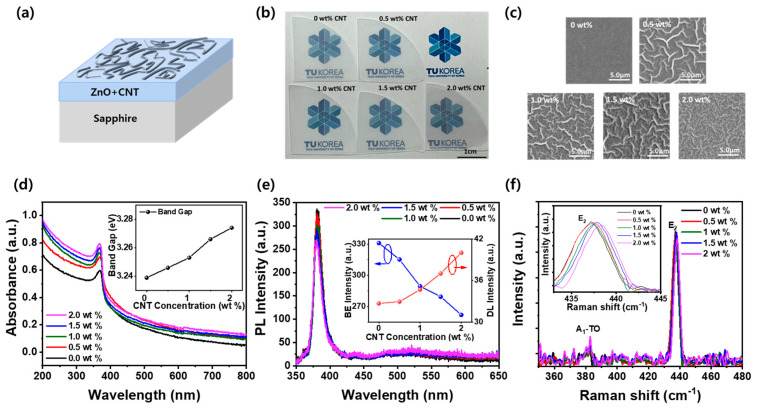
(**a**) Schematic diagram of ZnO–CNT nanocomposite thin film with surface wrinkle structures. (**b**) Photographic image, (**c**) SEM images, (**d**) UV-visible absorption spectra, (**e**) PL spectra, and (**f**) Raman spectra of ZnO–CNT nanocomposite films with different CNT contents (0–2.0 wt%). The insets of (**d**–**f**) show the variation in bandgap energy, the intensity ratio of NBE to DLE, and the E_2_ peak position as a function of CNT concentration, respectively.

Figure 1a shows a schematic illustration of the ZnO–CNT thin film structure fabricated via a sol–gel process on sapphire substrate. The diagram highlights the formation of surface wrinkle structures, which are induced by the incorporation of CNTs into the ZnO matrix. These wrinkles reflect strain effects and localized morphological distortions caused by CNT dispersion during the sol–gel and annealing processes [28]. Figure 1b displays a photographic image of quarter-sapphire wafers (1-inch radius) coated with ZnO–CNT nanocomposite thin films at varying CNT concentrations (0~2.0 wt%). The films with 0–1.5 wt% CNT appear nearly transparent, indicating uniform dispersion and good optical clarity. However, at 2.0 wt% CNT, a slight darkening is observed, attributed to CNT aggregation, which reduces transparency and suggests a threshold beyond which uniform nanocomposite formation becomes limited. Figure 1c presents SEM images of the surface morphologies of ZnO–CNT films with varying CNT concentrations. In the CNT-free ZnO film, a relatively smooth surface without wrinkle formation was observed. However, when 0.5 wt% CNT was incorporated, nanowrinkles with an average width of approximately 350 nm began to form. As the CNT content increased, the wrinkle width also increased, reaching nearly 900 nm at 2.0 wt% CNT, forming broader and more relaxed wrinkle structures. This evolution in surface morphology is attributed to buckling induced by compressive stress, which arises during the low-temperature solvent evaporation step of the sol–gel process, performed at 150 °C for 5 min. The incorporation of CNTs modulates the stress distribution in the thin film, promoting the formation of characteristic wrinkle structures due to differential shrinkage and elastic mismatch between the CNT network and the ZnO matrix. To examine the optical properties of ZnO–CNT thin films, UV-visible absorption spectra were measured as a function of CNT content, as shown in Figure 1d. As the CNT concentration increased, the overall absorbance increased across the entire wavelength range, indicating enhanced light absorption due to the intrinsic optical characteristics of CNTs and increased light scattering from the roughened film surface [29]. In addition, the absorption edge exhibited a clear blueshift, with the optical bandgap estimated from Tauc plots increasing from 3.239 eV (0 wt%) to 3.274 eV (2.0 wt%), as shown in the inset of Figure 1d. This bandgap widening is attributed to compressive stress induced by CNT incorporation, which leads to lattice distortion in the ZnO matrix and modifies its electronic band structure. Such stress arises during the sol–gel processing and thermal annealing stages, particularly due to the mismatch in thermal and mechanical properties between ZnO and CNTs. The resulting nanowrinkle structures observed in Figure 1c further support this interpretation, as the wrinkle formation is a direct manifestation of stress-driven buckling, which in turn modifies the electronic structure and shifts the optical bandgap [30]. Figure 1e shows the photoluminescence (PL) spectra of ZnO–CNT composite thin films, revealing a dominant near-band-edge (NBE) emission peak around 3.3 eV and a broad deep-level emission (DLE) band from 2.0 to 2.8 eV [31]. All samples exhibit strong NBE emission and weak DLE, indicating high optical quality; however, as shown in the inset of Figure 1e, the intensity of the NBE emission decreases, while the DLE intensity increases with increasing CNT content. This suggests enhanced defect formation, such as oxygen vacancies, interstitial zinc, and CNT-induced local distortions, due to interfacial stress and lattice mismatch during the sol–gel and annealing processes. These native and interfacial defects not only act as non-radiative recombination centers but are also known to influence the optical bandgap. In general, increased defect density can lead to either narrowing or widening of the bandgap, depending on the defect type, distribution, and associated strain field. In addition, the NBE peak position exhibits a slight blueshift with increasing CNT content, which is consistent with the optical bandgap widening observed in the inset of Figure 1d [32,33]. This blueshift behavior further supports the combined effect of compressive stress and defect-mediated band structure modification in the ZnO–CNT matrix. Figure 1f shows the Raman spectra of ZnO–CNT nanocomposite thin films with varying CNT concentrations. Characteristic phonon modes of ZnO, including the A_1_(TO) and E_2_(high) modes, are clearly identified in all samples, indicating that the wurtzite crystal structure is well maintained regardless of CNT content [34]. As shown in the inset of Figure 1f, the E_2_(high) mode systematically shifts from 437.24 cm^−1^ at 0 wt% CNT to 437.81 cm^−1^ at 2.0 wt% CNT with increasing CNT content. This blueshift (anti-Stokes shift) of the Raman peak is typically attributed to the buildup of compressive stress within the ZnO lattice, which likely originates from interfacial strain and thermal expansion mismatch between the ZnO matrix and the embedded CNTs [35]. This lattice compression observed in Raman analysis correlates well with the blueshift of the optical bandgap and PL emission peaks in Figure 1d and Figure 1e, respectively—both of which further support the presence of compressive stress and band structure modulation in the ZnO–CNT films. Additionally, the nanowrinkle morphology shown in Figure 1c is consistent with compressive in-plane stress generated during solvent evaporation and film shrinkage in the sol–gel process, suggesting that the mechanical deformation and optical/electronic properties share a common origin in CNT-induced stress fields.

### 3.2. Electrical and Photoresponse Characteristics of Al/ZnO–CNT/Al Devices Under Dark and UV-Stimulated Conditions

**Figure 2:**
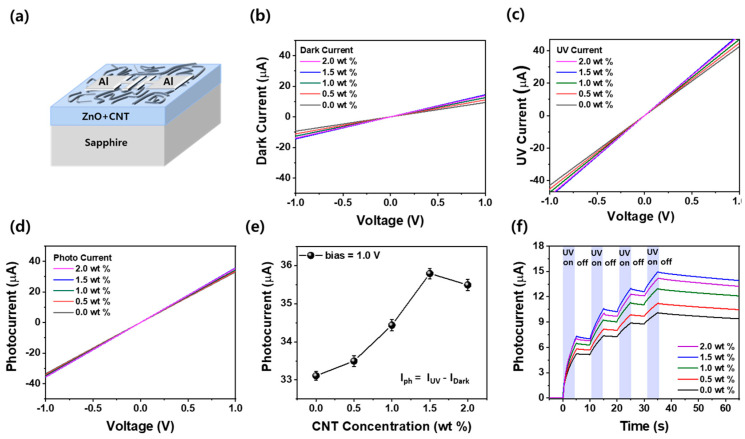
(**a**) The schematic diagram of the Al/ZnO–CNT/Al device. Current–voltage (I–V) characteristics of Al/ZnO–CNT/Al devices under (**b**) dark and (**c**) UV illumination conditions for various CNT concentrations. (**d**) Photocurrent extracted by subtracting dark current from UV current (I_ph_ = I_UV_ − I_dark_) at 1.0 V. (**e**) The photocurrent obtained at 1.0 V as a function of the CNT concentration in ZnO–CNT film. (**f**) Time-dependent photocurrent response under four cycles of UV on/off (5 s on, 5 s off).

Figure 2a shows a schematic of Al/ZnO–CNT/Al deposited on a sapphire substrate. Figure 2b presents the current-voltage (I-V) characteristics of the Al/ZnO/Al devices measured under dark conditions. The devices were fabricated on sapphire substrates with ZnO–CNT thin films prepared using the sol–gel process. Under dark conditions, the current at 1.0 V increased from 9.4 µA to 14.4 µA as the CNT content increased up to 1.5 wt%, indicating enhanced conductivity due to the CNT network [36]. However, at 2.0 wt% CNT, the current slightly decreased to 13.8 µA, which is likely due to excessive CNT aggregation that introduces scattering centers and obstructs carrier transport. Figure 2c shows the I–V curves of the same devices under 365 nm UV illumination. Compared to the dark condition, a steeper slope and high current values were observed, confirming a strong photoresponse. This enhancement is attributed to the generation of electron–hole pairs upon UV excitation and the delayed recombination of photogenerated carriers, particularly due to the trap states and charge separation effects enhanced by CNTs [26]. To quantify the photoresponse, Figure 2d displays the net photocurrent, defined as the difference between the UV and dark current (I_photocurrent_ = I_UV_ − I_dark_) of the Al/ZnO–CNT/Al optoelectronic synaptic device as a function of the applied voltage ranging from −1.0 V to +1.0 V. At 1.0 V, this difference is further compared in Figure 2e. As the CNT content increased, the photocurrent increased from 33.1 µA (0 wt%) to 35.8 µA (1.5 wt%) and then slightly decreased to 35.5 µA at 2.0 wt %. This trend suggests that moderate CNT incorporation enhances the photoconductive response by facilitating carrier transport and separation, while excessive CNT content may induce carrier recombination or percolation saturation, thereby reducing overall efficiency. Figure 2f shows the time-dependent photocurrent behavior of Al/ZnO–CNT/Al devices under repeated UV stimulation at 1.0 V. Each UV pulse was applied for 5 s and turned off for 5 s, repeated four times. Upon UV irradiation (UV on), all devices showed a rapid increase in photocurrent, attributed to the generation of electron–hole pairs in the ZnO layer. The incorporation of CNTs further facilitates carrier transport by forming a conductive network and promoting interfacial charge separation through p–n heterojunction formation with n-type ZnO. When the UV light was turned off (UV off), the photocurrent did not drop sharply but decayed gradually, indicating a PPC effect. This behavior arises from the trapping of photoinduced carriers at defect states and the CNT-ZnO interface, which delays recombination. 

Notably, with each cycle, the EPSC further increased, and the decay after UV off became slower, demonstrating a form of short-term synaptic potentiation through residual carriers—a hallmark of plasticity. As CNT content increased, both photocurrent amplitude and retention improved up to 1.5 wt%, owing to optimized CNT dispersion, effective light absorption, and enhanced carrier dynamics. However, at 2.0 wt% CNT, the photocurrent slightly decreased, and decay accelerated. This is likely due to CNT aggregation, which reduces active ZnO contact area, increases recombination sites, and interrupts uniform carrier pathways. Moreover, attempts to incorporate CNTs beyond 2.0 wt% led to further performance degradation, primarily due to poor dispersion and process limitations. Excessive CNT content impairs the wettability of the sol–gel solution during spin coating, resulting in non-uniform film spreading and partial delamination. These morphological instabilities limit reliable device fabrication and contribute to reduced optoelectronic performance. These findings highlight the suitability of ZnO–CNT composites, particularly at 1.5 wt% CNT, for efficient optoelectronic synaptic device applications by optimizing charge separation and carrier stability.

### 3.3. Photoresponse and PPF Characteristics of Al/ZnO–CNT/Al Optoelectronic Synaptic Devices

**Figure 3:**
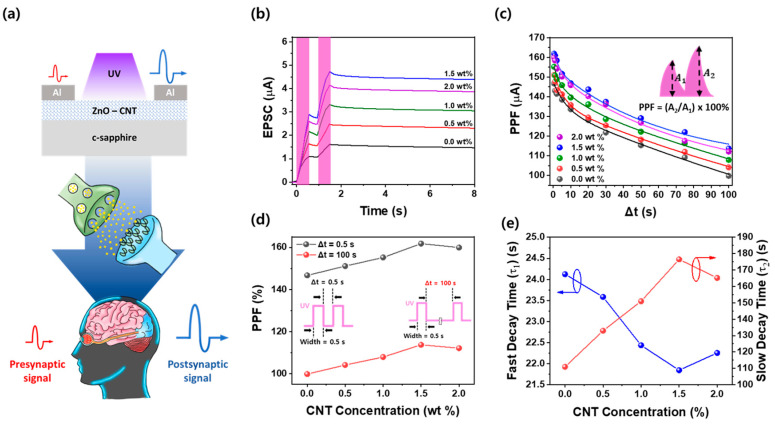
(**a**) Schematic of the Al/ZnO–CNT/Al optoelectronic synaptic device showing UV-induced EPSC generation, mimicking a biological synapse. (**b**) PPF responses under two consecutive UV pulses, showing enhanced second EPSC due to residual carriers. (**c**) PPF index vs. inter-pulse interval (Δt), with the highest PPF at Δt = 0.5 s and strongest facilitation at 1.5 wt% CNT. (**d**) PPF values at Δt = 0.5 s and 100 s as a function of CNT concentration in ZnO–CNT film. (**e**) Fast (τ_1_) and slow (τ_2_) decay times extracted by fitting the PPF vs. Δt curves in (**c**) using a double-exponential decay model.

Figure 3a illustrates a conceptual diagram of the Al/ZnO–CNT/Al optoelectronic synaptic device, where UV light serves as a presynaptic stimulus that generates a postsynaptic signal (EPSC) through photogenerated carrier dynamics in the ZnO–CNT layer. Upon UV irradiation, electron–hole pairs are generated in ZnO, and the p–n heterojunction formed with p-type CNTs facilitates efficient charge separation, leading to an enhanced EPSC. To evaluate the short-term synaptic plasticity characteristics of the ZnO–CNT nanocomposite optoelectronic synaptic devices, paired-pulse facilitation (PPF) experiments were performed under UV light stimulation. Figure 3b presents the EPSC responses of the devices to two consecutive UV pulses, each with a width of 0.5 s and a 50% duty cycle. The applied voltage was fixed at 1.0 V for all measurements. As CNT content increases, the EPSC improves, reaching a maximum at 1.5 wt%, where optimal charge separation and transport pathways are achieved. However, at 2.0 wt% CNT, the EPSC slightly decreases due to CNT aggregation, which introduces recombination sites and impedes uniform transport. In this setup, the first UV pulse induces a baseline EPSC (denoted as A_1_), followed by a second EPSC (A_2_) in response to the second pulse. The PPF index was calculated using the following equation [37,38]:

PPF = (A_2_/A_1_) × 100% (1)

This index quantifies the degree of facilitation observed between the two pulses. A higher PPF value indicates that the second pulse induces a significantly larger EPSC than the first, which is a hallmark of short-term plasticity. In all devices, the second EPSC was greater than the first, confirming the presence of a PPF effect. This enhancement is attributed to the accumulation of residual photogenerated carriers from the first stimulus that is still present when the second pulse arrives. These carriers assist in generating a stronger response to the second stimulus due to reduced recombination and delayed relaxation, akin to the facilitation observed in biological neural networks [37,38]. As the CNT content increased from 0 wt% to 1.5 wt%, both A_1_ and A_2_ increased, resulting in a higher PPF ratio. This trend reflects the improved charge separation and carrier mobility facilitated by the formation of a p–n heterojunction between the n-type ZnO and p-type CNTs. The heterojunction enhances interfacial electric fields, which assist in a more efficient extraction of photogenerated carriers, leading to stronger EPSC responses. The 1.5 wt% CNT device displayed the most significant facilitation, with a sharp rise in A_2_ relative to A_1_. This indicates that CNTs at this concentration are well dispersed and form optimal percolation pathways for carrier transport. However, when the CNT content increased to 2.0 wt%, a slight reduction in PPF was observed. This is likely due to CNT aggregation, which disrupts the uniformity of the composite film and introduces carrier recombination centers, thereby diminishing the synaptic enhancement effect. Figure 3c explores the effect of the inter-pulse interval (Δt) on the PPF response. Here, Δt was varied from 0.5 s to 100 s, and the PPF index was measured at each time point. The results demonstrate that PPF decreases as Δt increases, which is consistent with the decay of residual carriers over time [39]. At a short Δt of 0.5 s, the residual carriers from the first pulse are still abundant when the second pulse is applied, resulting in maximal facilitation. The PPF index reaches its peak at this interval, particularly for the 1.5 wt% CNT device, where a value of 161.8% was recorded. As Δt increases, the contribution of the residual carriers diminishes due to recombination and relaxation processes, causing the PPF value to decay. At Δt = 100 s, the PPF value for the 1.5 wt% device is still maintained at 113.7%, whereas the pure ZnO device exhibits a much lower value of 99.8%, indicating little to no facilitation. Figure 3d further elaborates on the compositional dependence of PPF at two specific time intervals: Δt = 0.5 s and Δt = 100 s. At the shorter interval, the PPF value increases with CNT content, reaching a peak of 161.8% at 1.5 wt%, followed by a slight decline to 159.9% at 2.0 wt%. Similarly, at Δt = 100 s, the PPF values decrease across all samples, but the 1.5 wt% CNT device consistently maintains a higher facilitation index than the others. The pure ZnO device displays a PPF of 146.7% at 0.5 s, which is considerably lower than that of the 1.5 wt% device, highlighting the positive role of CNTs in reinforcing the synaptic response. This trend confirms that an optimal CNT concentration exists, where the trade-off between carrier mobility enhancement and defect-induced recombination is minimized. 

Figure 3e presents the variation in fast (τ_1_) and slow (τ_2_) decay time constants of the PPF response concerning CNT concentration, derived by fitting the PPF decay curve using the dual-exponential model [40]:

PPF(t) = A_1_exp(−t/τ_1_) + A_2_exp(−t/τ_2_) (2)

In this equation, τ_1_ corresponds to the fast decay component, reflecting the dynamics of free carriers or shallow trap states, which are likely associated with interstitial (Zni) or surface-adsorbed oxygen species. On the other hand, τ_2_ represents the slow decay process, which is attributed to deeper trap sites such as oxygen vacancies (Vo) and CNT-induced interfacial defects. These states contribute to prolonged charge retention by acting as long-lived recombination centers at the ZnO–CNT junction [40]. As the CNT content increases from 0 to 1.5 wt%, τ_1_ decreases from ~24.1 s to ~21.8 s, indicating faster initial relaxation due to improved charge transport through the CNT network. Conversely, τ_2_ steadily increases from ~110 s to a peak of ~177 s at 1.5 wt%, suggesting enhanced long-term retention through interfacial carrier trapping and recombination suppression. At 2.0 wt%, both parameters slightly deviate from the trend, likely due to CNT aggregation. These results confirm that 1.5 wt% CNT provides optimal synaptic behavior, enabling both rapid response and extended memory retention, essential for mimicking short-term plasticity in neuromorphic systems.

### 3.4. UV-Dependent Synaptic Plasticity of Al/ZnO–CNT/Al Devices Under Various Stimulation Conditions

**Figure 4:**
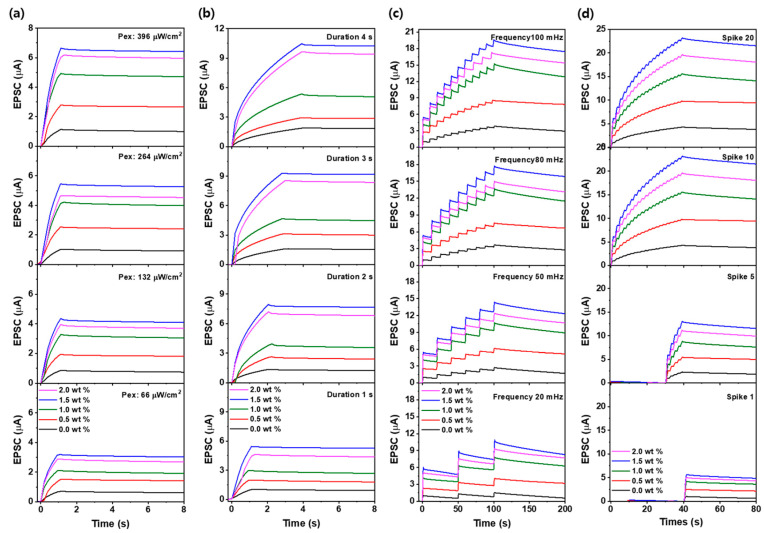
EPSC response and retention characteristics under varying (**a**) UV light intensity (66–396 μW/cm^2^), (**b**) pulse durations (1–4 s), (**c**) pulse frequencies (20–100 mHz), and (**d**) the number of UV pulses (1–20 spikes).

Figure 4a–d illustrate the EPSC responses and their decay behavior under various UV stimulation conditions, including intensity (66, 132, 264, 396 μW/cm^2^), duration (1.0–4.0 s), frequency (20–100 mHz), and number of spikes (1–20) [41]. The experiments were conducted on both ZnO-only and ZnO–CNT composite-based Al/ZnO–CNT/Al optoelectronic synaptic devices. Upon UV initiation, both device types exhibited an immediate increase in EPSC, followed by a slow decay after the cessation of the stimulus, indicating a PPC effect. This sustained EPSC behavior demonstrates synaptic-like memory characteristics. Notably, the device containing 1.5 wt% CNT consistently showed a higher EPSC and longer retention time compared to the ZnO-only counterpart under the same UV stimulation condition. Especially, while the ZnO-only device exhibited EPSC values ranging from 1.2 to 4.0 µA, the ZnO–CNT (1.5 wt%) composite device achieved significantly enhanced responses ranging from 6 to 23 µA. This improvement is attributed to the formation of a p–n heterojunction between p-type CNTs and n-type ZnO, which facilitates efficient electron–hole separation and suppresses recombination.

The resulting heterojunction structure promotes prolonged carrier lifetimes, thereby maintaining a higher EPSC over extended periods. From these results, we believe that CNT incorporation significantly enhances UV-induced synaptic plasticity, enabling stronger photoresponses and improved long-term memory behavior, which are essential for neuromorphic device applications.

### 3.5. CNT Fraction-Dependent Modulation of Learning, Forgetting, and Energy-Efficient Synaptic Plasticity in ZnO–CNT Optoelectronic Devices

**Figure 5:**
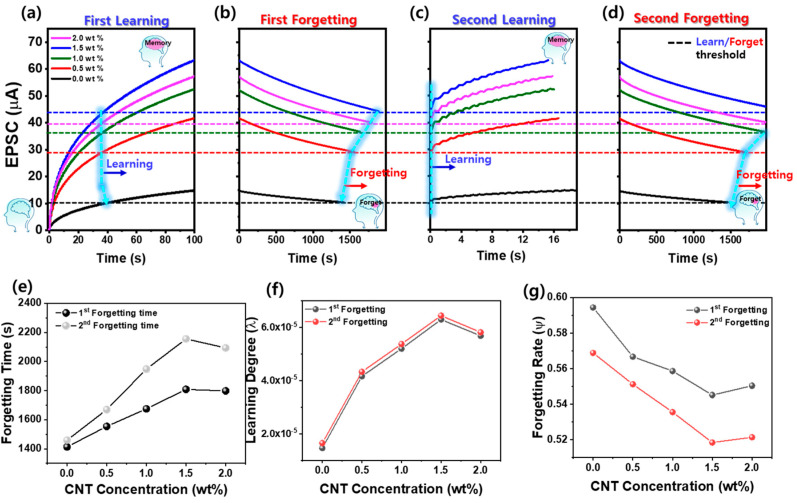
Learning–forgetting experience-dependent modulation of synaptic behavior in ZnO–CNT optoelectronic devices with different CNT concentrations: (**a**) first learning process under repeated UV pulses, (**b**) first forgetting showing EPSC decay after UV termination, (**c**) second learning process initiated from the forgetting threshold, and (**d**) second forgetting process. (**e**) Comparison of forgetting time for the first and second cycles. (**f**) Learning degree (λ) and (**g**) forgetting rate (Ψ), extracted from EPSC decay, are both modulated by CNT concentration. The cyan-colored lines in (**a**–**d**) represent the variation in the time required to reach the learning or forgetting threshold levels (black (0 wt%), red (0.5 wt%), green (1.0 wt%), blue (1.5 wt%), and magenta (2.0 wt%) dotted lines), defined as 70% of the maximum EPSC, as a function of CNT concentration.

Figure 5a–d present a systematic investigation of the learning–forgetting experience behavior of ZnO–CNT nanocomposite optoelectronic synaptic devices with varying CNT content. As the CNT concentration increased from 0 wt% to 2.0 wt%, the device exhibited enhanced synaptic plasticity, with the EPSC rising progressively in response to repeated UV stimuli, especially peaking at 1.5 wt%. The learning behavior was characterized by a gradual EPSC increase upon application of 100 UV light pulses (pulse width: 0.5 s, duty cycle: 50%, power density: 396 µW/cm^2^). To assess energy efficiency, the optical energy consumed per UV pulse (E) was calculated using the following equation [42]:

E = P × A × t × α(3)

where P is the light output density (396 µW/cm^2^), A is the illumination region (channel length: 50 µm × width: 825 µm), t is the pulse duration (0.5 s), and α is the ZnO absorption rate (0.20). Using this equation, the absorbed energy per UV pulse was estimated to be approximately 16.34 nJ. Although this energy level is slightly higher than that of biological synapses (typically ~10 fJ to a few nJ per event), the relatively large device size used in this study suggests that miniaturization could enable ultra-low power operation, bringing energy consumption into the biologically relevant regime. The forgetting process was initiated by UV termination, and the decay of EPSC was monitored over time. This learning–forgetting cycle was repeated twice to evaluate the temporal dynamics and retention behavior across different CNT concentrations. During the first learning phase (Figure 5a), the EPSC increased gradually under 100 UV pulses and reached its peak at 1.5 wt% CNT, indicating the strongest synaptic potentiation. In addition, the number of UV pulses required to reach the maximum EPSC in the first cycle gradually decreased with increasing CNT content, highlighting the role of the CNTs in accelerating synaptic strengthening. The maximum EPSC achieved at each CNT concentration, which gradually increased with CNT content and reached a saturation value of 63.3 μA for 1.5 wt% CNT, is also included in Figure 5a. In the first forgetting phase (Figure 5b), the time required for the EPSC to decay to 70% of its peak value increased with CNT content, reaching a maximum of 1810 s at 1.5 wt%, indicating stronger memory retention due to enhanced charge trapping and suppressed recombination. The forgetting times, which correspond to each CNT content, are also plotted in Figure 5e. The second learning phase (Figure 5c) began from the forgetting threshold levels (blue, magenta, green, red, and black dot lines), and the number of pulses required to recover maximum EPSC decreased with increasing CNT content. This trend, which demonstrates improved relearning efficiency due to residual carrier accumulation, is also reflected in the CNT-dependent learning pulse comparison shown in Figure 5a,c. As shown in Figure 5d, the second forgetting phase also exhibited extended memory retention, similar to the first forgetting cycle. Moreover, as shown in Figure 5e, the forgetting time after the second learning cycle increased for all CNT concentrations compared to the first cycle. In particular, the ZnO-only device showed an increase of approximately 60 s (1460 s), while the 1.5 wt% CNT device showed a 345 s increase, reaching 2155 s, confirming that repeated learning enhances long-term memory retention. This forgetting time (1400–2155 s) is notably longer than those of many previously reported optoelectronic synaptic devices based on materials MoS_2_, Ga_2_O_3_, CNTs, ZnO, and perovskite, which typically range from 10 to 900 s [37,38,39,41,42,43,44,45]. Such extended retention is attributed to the combined effects of persistent photoconductivity and CNT-induced deep-level trap states that suppress recombination and prolong charge storage. These results collectively demonstrate that both short-term and long-term plasticity in ZnO–CNT optoelectronic synaptic devices can be finely tuned by adjusting CNT concentration and that iterative optical stimulation significantly strengthens memory characteristics through cumulative charge retention and persistent photoconductivity enhancement. Furthermore, based on the learning and forgetting response curves shown in Figure 5a–d, Wickelgren’s power law was employed to quantitatively analyze the forgetting behavior of the Al/ZnO–CNT/Al optoelectronic synaptic devices. This model, widely used to describe biological memory decay, is expressed in the following Equation [46,47]:

I = λ × (1 + βt)^−Ψ^ (4)

where I denotes the memory intensity, t is the decay time, λ represents the initial learning degree (or the long-term memory state at t = 0), β is the scaling parameter, and Ψ is the forgetting rate that reflects how rapidly the memory fades. Figure 5f,g present the extracted fitting parameters, λ and Ψ, from the power-law model as a function of CNT concentration. As shown in Figure 5f, the learning degree (λ) increases with CNT content in both the first and second learning cycles, reaching a maximum at 1.5 wt%. This trend confirms that CNT incorporation facilitates more efficient charge accumulation and stronger initial synaptic responses due to enhanced carrier separation and transport.

Figure 5g shows the corresponding forgetting rate (Ψ), which systematically decreases with increasing CNT content, with the lowest values observed at 1.5 wt%. This reduction in Ψ indicates slower EPSC decay and improved synaptic memory stability. Notably, the second forgetting cycle exhibits consistently lower Ψ values than the first across all CNT concentrations, reflecting the cumulative memory reinforcement effect of repeated training. These quantitative analyses support the experimental EPSC results shown in Figure 5a–d, demonstrating that CNT incorporation significantly enhances both memory formation and long-term retention in ZnO–CNT optoelectronic synaptic devices.

### 3.6. Visual Memory Simulation and Retention Analysis of ZnO–CNT Synaptic Device Array

**Figure 6:**
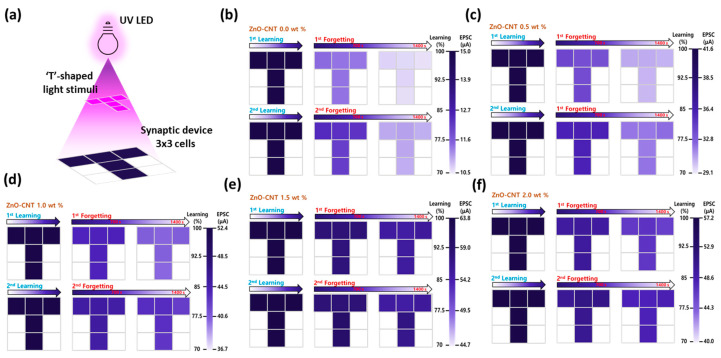
Visual memory simulation of ZnO–CNT optoelectronic synaptic devices under patterned UV stimulation: (**a**) Schematic of the selective UV stimulation process used to project a T-shaped pattern onto a 3 × 3 array of ZnO–CNT synaptic devices. Learning and forgetting behavior of devices with different CNT concentrations: (**b**) 0 wt%, (**c**) 0.5 wt%, (**d**) 1.0 wt%, (**e**) 1.5 wt%, and (**f**) 2.0 wt%. The measured EPSC values are mapped to pixel intensity and color to encode the optical memory state across the array.

To express visual memory, selective optical stimulation was applied to an array of ZnO–CNT optoelectronic synaptic devices. As illustrated in Figure 6a, a T-shaped UV light pattern was projected on a 3 × 3 pixel array, simulating spatially resolved memory formation. Each pixel in the array corresponds to an individual Al/ZnO–CNT/Al synaptic device with a channel area of 50 μm × 825 μm, ensuring uniform geometry and optical input across the array. Figure 6b–f presents the corresponding visual memory states for devices with different CNT concentrations (0–2.0 wt%), allowing the evaluation of both spatial uniformity and memory retention performance across the device matrix [45]. Each synaptic pixel device was stimulated with 100 UV light pulses (pulse width: 0.5 s, duty cycle: 50%), and the resulting EPSC values were converted to color intensities, where darker pixels represent stronger EPSC responses, i.e., more robust memory states. This color mapping effectively visualized the learning and forgetting behavior through the evolution of the T-shaped memory pattern. In the first learning–forgetting cycle, noticeable differences in retention were observed depending on CNT concentration. After 1400 s, the 0 wt% CNT device exhibited substantial EPSC decay, resulting in faded, lighter-colored pixels, whereas the 1.5% wt% CNT device maintained darker hues, indicating stronger memory retention. This suggests that moderate CNT content enhances carrier confinement and suppresses recombination, thereby improving memory stability.

In the second learning–forgetting cycle, a similar trend was observed across all CNT-containing devices, but with notably enhanced retention, particularly for 1.5 wt% CNT configuration. This improved performance reflects memory reinforcement effects due to repetitive stimulation, mimicking biological memory consolidation. These visual simulations demonstrate that synaptic learning and forgetting characteristics can be systematically modulated by controlling the CNT content in the ZnO–CNT nanocomposite film. Such material-level control enables the realization of neuromorphic visual memory devices with high stability, low power consumption, and extended memory retention, underscoring the importance of composite engineering in optimizing artificial synaptic device functionality.

## 4. Conclusions

In this study, ZnO–CNT nanocomposite-based optoelectronic synaptic devices were successfully fabricated using a sol–gel method and evaluated for their synaptic plasticity under various ultraviolet (UV) stimulation conditions. By varying the CNT content from 0 to 2.0 wt%, the electrical, optical, and neuromorphic performance of the devices was systematically investigated. The incorporation of CNTs significantly enhanced EPSC response, memory retention, and learning efficiency due to the formation of p–n heterojunctions, which facilitated efficient electron–hole separation and suppressed recombination. Among all compositions, the 1.5 wt% CNT device exhibited the best performance in terms of photocurrent generation, paired-pulse facilitation, long-term memory retention, and learning–forgetting reproducibility. Energy consumption per UV pulse was calculated to be 16.34 nJ, which can be further reduced through device miniaturization. A visual memory simulation using a 3 × 3 synaptic array demonstrated stable and programmable memory states, clearly showing that CNT content can regulate synaptic behavior in a spatially controlled manner. These findings highlight the potential of ZnO–CNT composite materials for energy-efficient, stable, and tunable neuromorphic systems, offering a promising route toward hardware-based artificial intelligence. The material-level strategy proposed here provides a foundation for designing multifunctional optoelectronic synaptic devices with enhanced learning and memory capabilities.

## Data Availability

The data presented in this study are available upon request from the corresponding author. The data are not publicly available due to privacy concerns.
